# Elucidating the Role of Transcriptomic Networks and DNA Methylation in Collagen Deposition of Dezhou Donkey Skin

**DOI:** 10.3390/ani14081222

**Published:** 2024-04-18

**Authors:** Xinrui Wang, Wei Ren, Yongdong Peng, Muhammad Zahoor Khan, Huili Liang, Yigang Zhang, Xiaotong Liu, Yinghui Chen, Xiyan Kou, Liyuan Wang, Changfa Wang, Yandong Zhan

**Affiliations:** Liaocheng Research Institute of Donkey High-Efficiency Breeding and Ecological Feeding, Liaocheng University, Liaocheng 522000, China2110190109@stu.lcu.edu.cn (W.R.); pengyongdong@lcu.edu.cn (Y.P.); zahoorcau@cau.edu.cn (M.Z.K.);

**Keywords:** DNA methylation, transcriptome, collagen deposition, Dezhou donkey, *COL1A1*

## Abstract

**Simple Summary:**

This study investigates the impact of DNA methylation on collagen deposition in the skin of Dezhou donkeys, a breed valued for its thick, flexible skin with medicinal properties. Utilizing whole genome bisulfite sequencing (WGBS) and RNA sequencing (RNA-seq), the research analyzes the epigenetic landscape and gene expression profiles across three developmental stages of Dezhou donkeys. The study identifies numerous differentially methylated genes related to collagen deposition, such as *COL1A1*, *COL1A2*, and *MMPs*, highlighting an inverse relationship between gene expression and DNA methylation near transcription start sites. The findings of our study reveal the significant regulatory role of DNA methylation in skin collagen deposition, offering insights for genetic improvement and selective breeding to enhance skin quality in Dezhou donkeys. Our current research adds to the foundational knowledge of collagen deposition mechanisms, contributing to the fields of molecular biology and animal husbandry.

**Abstract:**

DNA methylation represents a predominant epigenetic modification with broad implications in various biological functions. Its role is particularly significant in the process of collagen deposition, a fundamental aspect of dermal development in donkeys. Despite its critical involvement, the mechanistic insights into how DNA methylation influences collagen deposition in donkey skin remain limited. In this study, we employed whole genome bisulfite sequencing (WGBS) and RNA sequencing (RNA-seq) to investigate the epigenetic landscape and gene expression profiles in the dorsal skin tissues of Dezhou donkeys across three developmental stages: embryonic (YD), juvenile (2-year-old, MD), and mature (8-year-old, OD). Our analysis identified numerous differentially methylated genes that play pivotal roles in skin collagen deposition and overall skin maturation, including but not limited to *COL1A1*, *COL1A2*, *COL3A1*, *COL4A1*, *COL4A2*, *GLUL*, *SFRP2*, *FOSL1*, *SERPINE1*, *MMP1*, *MMP2*, *MMP9*, and *MMP13*. Notably, we observed an inverse relationship between gene expression and DNA methylation proximal to transcription start sites (TSSs), whereas a direct correlation was detected in regions close to transcription termination sites (TTSs). Detailed bisulfite sequencing analyses of the *COL1A1* promoter region revealed a low methylation status during the embryonic stage, correlating with elevated transcriptional activity and gene expression levels. Collectively, our findings elucidate key genetic markers associated with collagen deposition in the skin of Dezhou donkeys, underscoring the significant regulatory role of DNA methylation. This research work contributes to the foundational knowledge necessary for the genetic improvement and selective breeding of Dezhou donkeys, aiming to enhance skin quality attributes.

## 1. Introduction

The Dezhou donkey is recognized as one of the five important donkey breeds in China [1], renowned for its thick, flexible skin, which is not only valued for leather production but also possesses medicinal properties. Notably, the dermal collagen is the primary ingredient in Ejiao, a revered traditional Chinese medicine known for its blood-enriching, immunomodulatory, and anti-aging benefits [2,3,4]. However, as the demand for Ejiao increases, the demand for donkey hides also increases sharply. With the advancement of science and technology, the labor function of donkeys has been weakened, and due to factors such as the long production cycle of donkeys and the slow progress in breeding improvement, the donkey population has declined, resulting in the scarcity of donkey skins in the Chinese market, requiring a large portion of donkey skins to be imported [5,6]. Consequently, enhancing growth rates to augment hide yield has emerged as a critical area of research in Dezhou donkey husbandry.

Collagen plays a crucial role in skin development. As the primary component of the dermal layer, it forms a fibrous network that provides robust structural support to the skin [7]. Additionally, collagen contributes to elasticity, wrinkle resistance, wound healing, moisturization, and protection [8]. By studying skin tissues at various developmental stages, we can gain a better understanding of the role of collagen in skin development and biological processes.

The evolution of science and technology has ushered in a transition from traditional breeding techniques to molecular ones in animal husbandry, with marker-assisted and genomic selection becoming the cornerstone of livestock molecular breeding [9]. RNA sequencing (RNA-seq), a pivotal tool in transcriptomics, plays a crucial role in correlating gene expression with phenotypic and functional attributes of animals [10], facilitating the identification of genes linked to complex traits [11,12]. Furthermore, RNA-seq enables the examination of dynamic gene expression changes and gene interactions [13]. Previous studies, such as those conducted by Chai et al., leveraged RNA-seq to pinpoint candidate genes implicated in enhancing meat quality in Dezhou donkeys, including *MYH1*, *MYH7*, *TNNC1*, *TNNI3*, *TPM3*, *ALDOA*, *ENO3*, and *PGK1* [14]. Other investigations have identified key genes related to skin thickness, reproductive traits, muscle development, and coat color in donkeys [15,16,17,18].

DNA methylation is a major epigenetic modification mechanism in eukaryotic organisms. It can influence gene expression and chromatin structure without altering the DNA sequence. This modification induces heritable changes in gene function [19] and impacts various biological processes, including tissue-specific gene expression, cell differentiation, genomic imprinting, and diseases [20]. This modification, mediated by DNA methyltransferases (DNMTs), involves the transfer of a methyl group to cytosine [21] and is reversible [22]. DNMT1 catalyzes methylation modifications on newly synthesized DNA strands, maintaining existing methylation states [23]. DNMT3A and DNMT3B, as functional enzymes with methyltransferase activity, can add methyl groups to newly synthesized DNA under the influence of histone modifications [24,25]. DNA methylation, a widely studied epigenetic modification, represents a relatively stable state of modification that can be inherited by subsequent generations of DNA. In the field of dermatology, epigenetic modifications play a crucial role in the occurrence and progression of autoimmune-related skin diseases [26]. Simultaneously, they also have found extensive applications in the field of livestock breeding, particularly in modulating fat deposition by regulating gene expression through the inhibition of transcription factor binding [27].

Contemporary research increasingly combines epigenetic and omic approaches to unravel the molecular regulatory mechanisms of differentially expressed genes. For instance, studies by Zhang et al. utilized DNA methylation and transcriptome sequencing technologies to identify differentially methylated regions (DMRs) and genes (DEGs) associated with intramuscular fat deposition in chickens [28]. Another study by Zhang et al. integrated genome-wide methylation and transcriptome analysis to investigate the methylation and gene expression profiles in goose leg muscle tissues, revealing that DNA demethylation of myogenic genes may contribute to variations in leg muscle development among embryonic geese [29].

As an original local breed, the Dezhou donkey lacks a systematic breeding process. Additionally, there is low intra-group uniformity in the collagen protein content of donkey skin. Furthermore, the gene expression and epigenetic mechanisms underlying collagen deposition during donkey skin development remain unclear. Therefore, investigating key factors such as gene expression and DNA methylation during the growth and development of donkey skin tissues, from embryonic stages to adulthood, is essential. Such analysis can help identify critical molecular regulatory mechanisms and lay the foundation for future research on donkey skin development and genetic breeding.

## 2. Materials and Methods

### 2.1. Ethical Statement

The experimental procedures regarding experiments and animals care were performed as per Animal Welfare and Ethics Committee of Institute of Animal Sciences, Liaocheng University under Ethical number (LC2019-1).

### 2.2. Animals and Sample Collection

The Dezhou donkeys used in this study were sourced from a breeding base in Dezhou City, Shandong Province, China. They had the same feeding conditions and breeding management conditions. The nutrition of the donkeys in the entire donkey farm was balanced with an abundant supply of clean water. The environment was well-ventilated and dry, and the donkeys were free from any diseases. Back skin tissue samples were collected from Dezhou donkeys at different stages: 8-month-old fetal stage (YD, *n* = 3), 2-year-old (MD, *n* = 3), and 8-year-old (OD, *n* = 3). All sampling sites were located in the middle of the left back, between the sixth and seventh thoracic vertebrae. The samples in the YD period were aborted after being squeezed by external force, and the skin sampling was completed within 1 h. During the MD and OD periods, sample collection was carried out through minimally invasive skin sampling. Before sampling, we used professional tools to prepare the skin of the sampling site, then used procaine to reduce pain, and finally used a 5 mm skin sampler (Acuderm, Fort Lauderdale, FL, USA) to collect samples. The collected skin tissue samples were rinsed with PBS, quickly cooled in liquid nitrogen, and stored in a −80 °C refrigerator for subsequent analysis. All the donkeys in this study were healthy and had a good prognosis.

### 2.3. Sequencing and Analysis of Methylomes

We independently extracted genomic DNA from nine skin tissues collected at three different periods. DNA concentration and integrity were detected using a nanophotometer^®^ spectrophotometer (IMPLEN, Westlake Village, CA, USA) and Agarose Gel Electrophoresis, respectively. After passing the test, they were sent to Genedenovo Biotechnology Co., Ltd. (Guangzhou, China) for the construction of a bisulfite DNA library and whole-genome sequencing. Genomic DNA samples were fragmented into 100–300 bp by sonication (Covaris, Woburn, MA, USA) and purified with MiniElute PCR Purification Kit (QIAGEN, Germantown, MD, USA). Next, a single “A” nucleotide was added to the 3′ end and ligated with a methylation adapter. DNA fragments with adapters were bisulfite-converted by Methylation-Gold kit (ZYMO, Irvine, CA, USA). Then, the converted DNA fragments were sequenced by Illumina HiSeqTM2500 (Illumina, San Diego, CA, USA). Finally, the raw reads were filtered to remove reads containing more than 10% unknown nucleotides and low-quality reads, thereby obtaining clean reads, which were used for subsequent analysis.

### 2.4. Methylation Data Analysis

The clean reads we obtained were mapped to the Dezhou donkey reference genome (ASM1607732v2) using BSMAP software (version: 2.90) [30] with default parameters. Pearson’s chi-square test (χ^2^) in methylKit (version: 1.7.10) [31] was employed to evaluate the methylation status and ratio of the genome-wide DNA methylation profiles. In the context of CG, when the GC number of each window was ≥5, the absolute value of the difference in methylation rate was ≥0.25, and the q was ≤0.05, we regarded these methylated regions as differential methylation regions. DMRs that overlapped with gene body, upstream 2 Kb, or downstream 2 Kb regions of the body were considered as differentially methylated genes (DMGs).

### 2.5. Sequencing and Processing of Transcriptome Data

In our study, we used the transcriptome data published by Wang, X. et al. (2024) as the basic data for this study [5]. The samples used for RNA sequencing were consistent with those used for whole genome bisulfite sequencing (WGBS). Total RNA from skin tissue was extracted using the Trizol reagent kit (Invitrogen, Carlsbad, CA, USA). After RIN was used to evaluate the quality of the RNA sample, it was sent to Genedenovo Biotechnology Co., Ltd. (Guangzhou, China) for RNA library construction and sequencing. After sequencing completed, we filtered the raw data to obtain clean reads for subsequent analysis. We then employed HISAT2.2.4 [32] to align the paired-end clean reads with the reference genome of the Dezhou donkey (ASM1607732v2). Finally, we normalized gene expression levels using Fragments Per Kilobase of exon model per Million mapped fragments (FPKM) values. This method effectively eliminates the influence of sequencing depth and gene length on gene expression levels, allowing for direct comparison of gene expression levels between different samples. This series of processing steps ensures the accuracy and reliability of our transcriptome data. Differential expression analysis was undertaken using DESeq2 [33] software (version: 1.20.0), which was employed to identify differentially expressed genes (DEGs) meeting the criteria of a fold change ≥2.00 and an adjusted *p*-value of 0.05.

### 2.6. Correlation of DMRs and DEGs between Groups

In order to investigate the possible roles that DNA methylation plays in DEGs, common genes between DMRs related genes and DEGs were analyzed. Additionally, gene ontology (GO) enrichment analysis and KEGG pathway enrichment analysis were conducted. DMGs were mapped to GO terms in the Gene Ontology database (http://www.geneontology.org/, accessed on 28 October 2023). Gene numbers were calculated for every term, and significantly enriched GO terms in genes compared to the genome background were defined by the hypergeometric test. KEGG annotation (http://www.genome.jp/kegg, accessed on 28 October 2023) was used to subject DMGs to KEGG enrichment analysis. False discovery rate (FDR) correction, specifically employing the Benjamini–Hochberg adjustment method, was applied. GO and KEGG terms boasting *p* values below 0.05 were identified as significantly enriched.

### 2.7. Bisulfite Sequencing PCR (BSP)

DNA methylation levels in *COL1A1* gene promoters were measured by the Bisulfite sequencing PCR (BSP). In total, 200 ng of the donkey skin tissue genomic DNA was treated with bisulfite. The DNA samples treated with sodium bisulfite were purified and recovered to ensure the acquisition of high-quality DNA. Then, we designed primers targeting the high CG content sequence in the promoter region of the *COL1A1* gene. The DNA was amplified using PCR. Finally, the PCR products were sent to Qingdao Biotech Co., Ltd. (Qingdao, China) for sequencing. BSP primers were designed by the Primer 5.0 software (Table 1). The methylation levels were visualized by MSRcall software (www.msrcall.com, accessed on 28 October 2023).

### 2.8. Statistical Analysis

Statistical analyses were performed using SPSS 26.0 software (Statistical Product and Service Solutions, Version 26.0 Edition, IBM, Armonk, NY, USA). In the present study, the results were presented as mean ± SEM and were subjected to statistical analysis by two-tailed *t*-test. The level of significance was presented as * *p* < 0.05 and ** *p* < 0.01.

## 3. Results

### 3.1. The DNA Methylation Atlas of Skin Tissues of Dezhou Donkey at Different Developmental Periods

**Figure 1:**
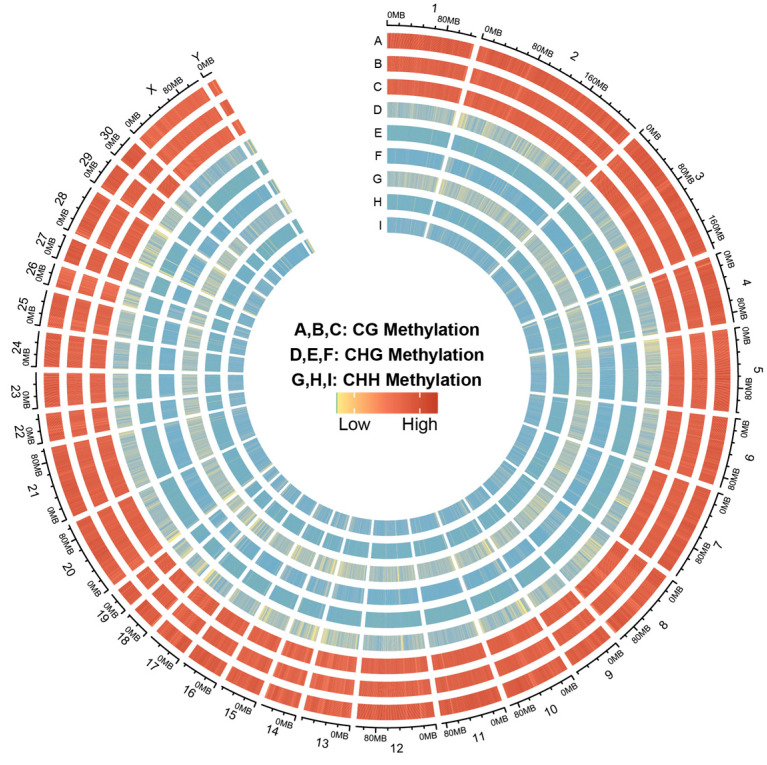
Distribution of identified methylation sites on each chromosome. The outer ring represents the Dezhou donkey genome labeled with chromosome number and position. (**A**–**C**) CG methylation; (**D**–**F**) CHG methylation; (**G**–**I**) CHH methylation; (**A**,**D**,**G**) YD period, (**B**,**E**,**H**) MD period, (**C**,**F**,**I**) OD period.

In the present study, 74.24, 72.13, and 75.52 G of raw data were generated in the skin tissues of Dezhou donkeys during the MD, OD, and YD periods, respectively. After removing the low quality, adapter, and reads containing more than 10% N, we finally acquired 518,959,330, 504,203,919, and 527,906,285 clean reads in MD, OD, and YD periods, respectively. Totals of 90.21%, 89.50%, and 90.28% of the Dezhou donkey genome were covered with the uniquely mapped reads in MD, OD, and YD periods, respectively. The Q20 value was more than 0.95; these results indicated a reliable sequencing outcome (Table 2). In addition, the Circos plot displayed the DNA methylation levels in the various sequence contexts (mCG, mCHG, and mCHH) (where H is A, C, or T) in Dezhou donkey chromosomes (1–30 and the X, Y chromosome; Figure 1).

### 3.2. Global DNA Methylation Patterns of Skin Tissues in Dezhou Donkey

**Figure 2:**
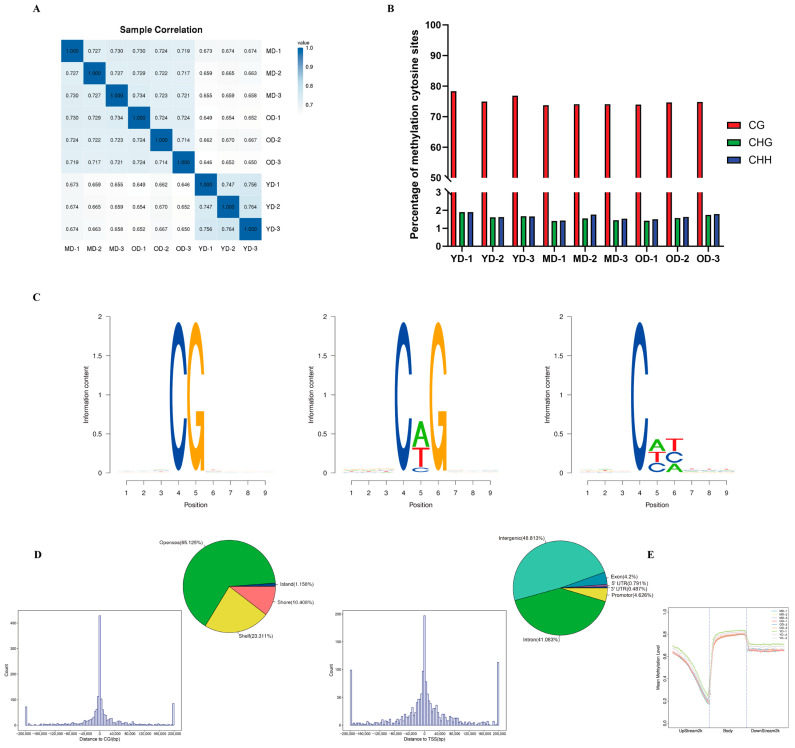
The DNA methylation characteristics of skin tissue in Dezhou donkeys. (**A**) The correlation analysis of the methylation between samples. (**B**) Comparison of DNA methylation patterns in different samples. (**C**) Sequence preferences for methylation in sequence contexts; 9 bp base information around the position of mCG, mCHG, mCHH at highest or lowest methylation levels, in which the methylation cytosine is in the fourth position. (**D**) The frequency distribution histogram of the distance from DMR to CGI and the distance from DMR to TSS. The DMR annotation in CGI function elements (island, shore, shelf, and open sea) and genome functional regions (5′UTR, 3′UTR, exon, intron, promoter, intergenic). (**E**) Average CG methylation levels in different function regions between different samples.

The analysis of Pearson correlation for the CpG base indicated that our samples exhibit strong data repeatability (r > 0.7) (Figure 2A). To investigate the differences of global DNA methylation profiles between the three groups, DNA methylation levels in three contexts—CG, CHG, and CHH (where H is A, C, or T)—were analyzed in the present study. The data shown in Figure 2B indicated that a majority of cytosines (75%) were methylated in the CpG context, whereas a relatively minor percentage (1.6%) were methylated in the GHG and CHH contexts (where H is A, C, or T). At the same time, in gene regulatory regions and transcriptional elements, CG context accounted for the highest proportion (62.40% on average), while CHG and CHH background methylation rates were low (1.6%) (Supplementary Table S1, Supplementary Figure S1). In order to investigate the methylation patterns of cytosines in Dezhou donkey skin tissue, we conducted an analysis of the genome-wide preferences for mC sequences in various sequence contexts. Based on our results, methylated cytosines were more often found in CG, CHG, and CHH (where H = A > T) (Figure 2C). Therefore, we focused on CG methylation patterns in this study. In the MD-vs.-OD group, the DMRs of the CGI were mainly located in the open sea (65.1%) and the shelf (23.3%). The DMRs were mainly located in the intergenic region (48.8%), followed by the introns (41.1%) and the promoter region (4.6%) (Figure 2D). The characteristics of the YD-vs.-MD and YD-vs.-OD groups are shown in Supplementary Figure S2. Moreover, the CG methylation levels of YD were higher than MD and OD periods. Additionally, we observed a dramatic decrease in DNA methylation in the TSS region, a sharp increase in the initial stage of gene body, and a plateau until the TTS (Figure 2E).

### 3.3. WGBS-Transcriptomic Data Integration

**Figure 3:**
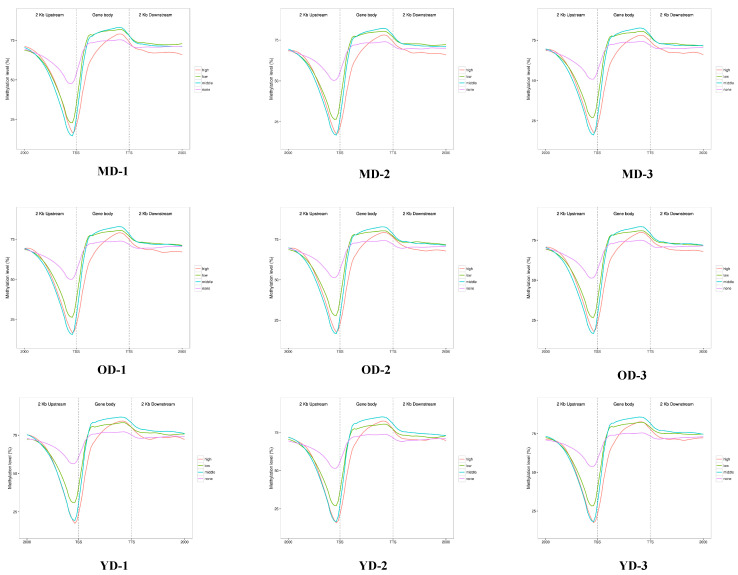
WGBS-transcriptomic data integration in gene regulatory regions in Dezhou donkey skin tissues at different developmental stages. According to RNA-seq data, genes were considered as differentially expressed (DEGs) if |log2(FC)| ≥ 1 and *p*-value ≤ 0.05. FPKM ≥ 100 was regarded as high expression; 10 ≤ FPKM < 100 was regarded as middle expression; 0 ≤ FPKM < 10 was regarded as low expression; FPKM = 0 was regarded as no expression. CG methylation levels in gene regulatory regions (upstream 2 Kb, gene body, and downstream 2 Kb) were analyzed in each group.

To learn more about how DNA methylation affects gene expression, the DNA methylation level (CG) of the gene regulatory region (upstream 2 Kb, gene body, and downstream 2 Kb) was detected. Based on RNA-seq data, genes were classified as high expression, middle expression, low expression, or no expression. The results in Figure 3 show that, in the upstream 2 Kb region, especially the region near the TSS, there is a negative correlation between gene expression levels and DNA methylation. The same results are also found in the downstream 2 Kb except for no expression. This suggests that higher gene expression in this region is associated with lower DNA methylation levels. In addition, except for the high expression group, positive correlations were found in the gene body close to TTS in the other expression groups. In summary, gene expression and DNA methylation levels in different gene regions are not always similarly correlated in Dezhou donkey skin tissues at different developmental stages.

### 3.4. Analysis of the Relationship between DEGs and DNA Methylation in Skin Tissues at Different Developmental Stages

**Figure 4:**
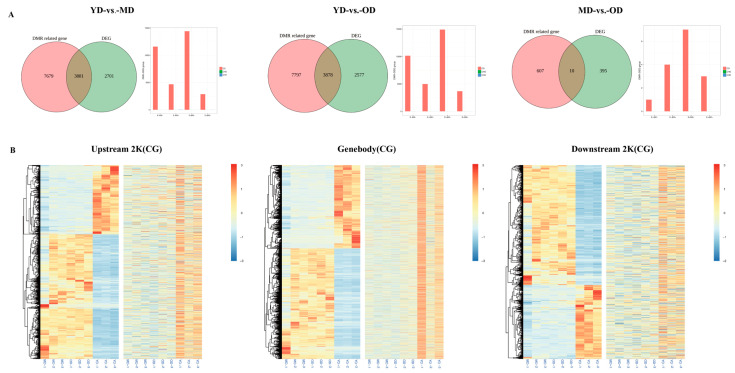
The identification of differentially expressed genes (DEGs) regulated by DNA methylation during skin development in Dezhou donkeys. (**A**) Venn diagram of DMR related genes and DEGs in YD-vs.-MD, YD-vs.-OD, and MD-vs.-OD groups together with statistical results of genes involved in different modes of DNA methylation and gene expression correlation. (**B**) Heatmap of gene expression patterns of genes that were both DMR-related genes and DEGs in upstream 2 Kb, gene body, and downstream 2 Kb regions (hierarchical cluster analysis on the left panel and non-hierarchical cluster analysis on the right panel). E, gene expression level; M, DNA methylation level; +, upregulation; −, downregulation.

To further identify genes regulated by DNA methylation during Dezhou donkey skin development, the DMR-regulated genes and DEGs in gene regulatory regions (upstream 2 Kb, gene body, and downstream 2 Kb) of Dezhou donkey skin were studied at three different stages in this work. In the YD-vs.-MD and YD-vs.-OD groups, the number of DMR-related genes and DEGs changed slightly, whereas in the MD-vs.-OD group, the number of DMR-related genes and DEGs was greatly reduced. There were 3881, 3878, and 10 genes that were both DMR-related genes and differentially expressed genes discovered in the three comparison groups of YD-vs.-MD, YD-vs.-OD, and MD-vs.-OD, respectively (Figure 4A). Among these three groups, a large number of genes were classified as upregulated expression and downregulated DNA methylation (E+&M−). In the YD-vs-MD group and the YD-vs.-OD group, downregulated expression and downregulated DNA methylation (E−&M−) was the second most common genotype, whereas in the MD-vs.-OD group, downregulated expression and upregulated DNA methylation (E−&M+) was the second most common genotype (Figure 4A).

Heatmaps of gene expression patterns were constructed to analyze the 7769 genes that were both DMR-related genes and DEGs (Figure 4B). In the clustering heat map, some genes in the YD period are higher in the upstream 2 K and gene body regions than in the MD and OD periods, but the results are the opposite in the downstream 2 K region. These results suggest that DEGs related to DNA methylation showed different expression and DNA methylation patterns during these three periods. The different expression patterns of these DMR-related differentially expressed genes may have different regulatory effects during skin development in Dezhou donkeys.

### 3.5. Enrichment Analysis of Methylation-Related DEGs

**Figure 5:**
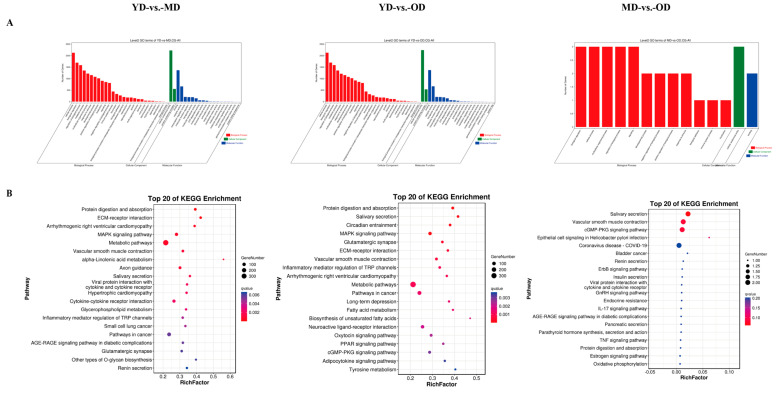
GO analysis and KEGG pathway analysis of DNA methylation-related DEGs. (**A**) GO analysis of DNA methylation-related DEGs in three groups (YD-vs.-MD, YD-vs.-OD, MD-vs.-OD). (**B**) KEGG pathway analysis of DNA methylation-related DEGs shows the top 20 KEGG enrichments in each comparison group.

To look into the biological functions of methylation-related DEGs, GO analysis and KEGG pathway analysis were conducted in three comparison groups. Our results indicated that the DMGs were mainly enriched in the cellular process, the biological regulation, the developmental process, the cellular anatomical entity, and the binding terms in the three comparison groups (Figure 5A). These biological processes play an important role in collagen deposition and skin health and function. The KEGG pathway analysis found some pathways related to collagen in the skin in the three comparison groups, such as ECM–receptor interaction, the MAPK signaling pathway, renin secretion, and the relaxin signaling pathway (Figure 5B).

### 3.6. Candidate DMGs Associated with Collagen in Dezhou Donkey Skin

**Figure 6:**
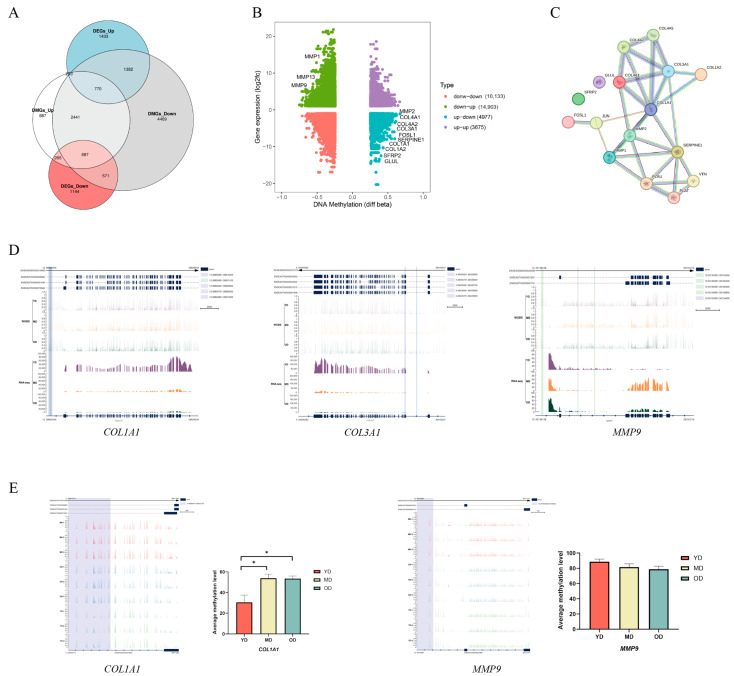
Candidate DMGs associated with collagen deposition. (**A**) The differential genes (DEGs) overlapped with differentially methylated genes (DMGs) in YD-vs.-OD group. (**B**) Integrated analysis of DNA methylation levels and gene expression levels. (**C**) Protein–protein interaction (PPI) network analysis of candidate DMGs associated with collagen deposition. (**D**) The DNA methylation levels (WGBS) and gene expression levels (RNA-seq) (IGV tracts) of three candidate DMGs (*COL1A1*, *COL3A1*, and *MMP9*). (**E**) The DNA methylation levels in the promoter region of candidate genes (*COL1A1* and *MMP9*). CpG regions around TSSs are marked in shadow area. Bar graph showing the average methylation level of gene at each period. * *p* < 0.05.

Although DMGs were found in the three comparison groups, the relevant mechanisms affecting collagen deposition and regulation in Dezhou donkey skin were unclear. Therefore, to explore whether candidate DMGs are related to collagen in Dezhou donkey skin, we integrated RNA-seq and WGBS data to reveal the methylated candidate genes related to collagen deposition and regulation in Dezhou donkey skin. In Figure 6A, there are 265 DMGs with high methylation levels and differential expression downregulation and 1382 DMGs with low methylation levels and differential expression upregulation during the collagen deposition and regulation process in the YD-vs.-OD group. We found several genes related to collagen deposition and degradation in skin, such as *COL1A1*, *COL1A2*, *COL3A1*, *COL4A1*, *COL4A2*, *GLUL*, *SFRP2*, *FOSL1*, *SERPINE1*, *MMP1*, *MMP2*, *MMP9*, and *MMP13* (Figure 6B). We also found genes related to collagen deposition and degradation in skin in the YD-vs.-MD group, but not in the MD-vs.-OD group (Supplementary Figure S3). Some genes were enriched in the ECM–receptor interaction, the relaxin signaling pathway, the MAPK signaling pathway, and the Hippo signaling pathway. Moreover, the protein–protein interaction (PPI) network analysis showed that the DMGs were highly correlated with each other (Figure 6C). The DNA methylation and gene expression levels of three DMGs, *COL1A1*, *COL3A1*, and *MMP9,* are shown in Figure 6D. In order to gain a deeper understanding of the expression patterns of collagen genes at different life stages, we analyzed and visualized the average DNA methylation levels near the transcription start sites (TSS) regions and the gene bodies of several collagen genes during three periods in Dezhou donkeys using IGV software (version: 2.3.26) (Figure 6E).

### 3.7. DNA Methylation of COL1A1 Promoter Region

**Figure 7:**
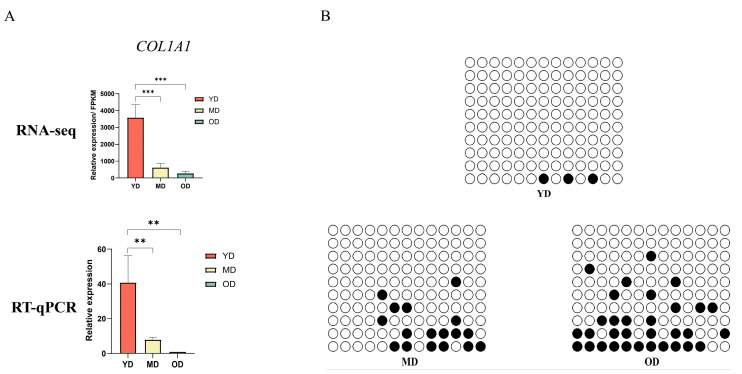
Validation of the association between DNA methylation and gene expression in the COL1A1 promoter region. (**A**) Gene expression of *COL1A1* gene in Dezhou donkeys during the three periods. (**B**) Pyrosequencing results of the 13 methylation sites within the promoter region of COL1A1 gene. ** *p* < 0.01 and *** *p* < 0.001.

Because *COL1A1* is essential for collagen deposition, DNA methylation patterns of the promoter and first exon regions at each stage from Dezhou donkeys were visualized by IGV software (version: 2.3.26) to analyze the correlation with *COL1A1* expression (Figure 6E). It was found that the average methylation level of the *COL1A1* gene in the promoter region was significantly higher in the MD and OD periods than in the YD period during the three periods. Combined with the *COL1A1* gene transcriptome level (Figure 7A), we speculated that the methylation of the *COL1A1* gene promoter region is negatively correlated with gene expression. Furthermore, pyrosequencing was performed to analyze DNA methylation levels in the *COL1A1* promoter region to validate the WGBS data (Figure 7B). The BSP results showed that, among the average methylation levels in the three periods, the average methylation level in the OD period was the highest, followed by the MD period, with the average methylation level in the YD period being the lowest, consistent with the WGBS results.

## 4. Discussion

Ejiao, recognized for its traditional medicinal properties in Chinese culture, plays a pivotal role in fortifying the body’s overall health and bolstering immunity. The cornerstone of Ejiao production lies in the extraction of collagen from donkey skin, a process heavily influenced by the concentration of collagen present. Recent studies have elucidated that the biodeposition of collagen is intricately regulated by specific genes [34]. Furthermore, an expanding body of research indicates that DNA methylation exerts a multifaceted influence on gene expression regulation. Despite the burgeoning interest in this field, the exploration of DNA methylation’s impact on gene transcription remains in its nascent stages [35], with a notable absence of such studies in donkey-specific research.

DNA methylation plays a key role in determining genome structure and function, including regulating cell differentiation and coordinating gene expression. In studies of human early embryonic development, gametes exhibited elevated levels of methylation in certain gene regions. During the embryonic stage, they maintained low methylation levels. In the zygote, parental methylation information was extensively erased, leading to the establishment of a novel methylation pattern. In mice and other mammals, similar DNA methylation dynamics occurred [36,37]. In studies related to different developmental stages in poultry, it was observed that DNA demethylation of myogenic genes may contribute to the differential leg muscle development in Wuzong geese and Shitou geese embryos [29]. This investigation embarked on a thorough examination of the DNA methylation patterns within the skin of the Dezhou donkey across various developmental stages. It posited that DNA methylation could significantly impact the deposition of collagen in donkey skin. The primary objective was to unearth methylated genes that could potentially influence the deposition of collagen within donkey skin.

The WGBS data revealed that, in this study, 75% of methylcytosines (mCs) were located within the CG context, with 1.6% in the CHG and CHH contexts. A notable pattern emerged showing a marked decrease in DNA methylation levels upstream of the TSS, a substantial increase across the gene bodies, and a stabilization towards the TTS. This pattern aligns with findings from other species, such as geese [29] and chickens [28]. Our analysis further demonstrated a negative correlation between gene expression and DNA methylation in regulatory regions proximal to TSS with a positive correlation observed near the TTS within gene bodies, corroborating findings in other species, such as pigs [38], humans [39], and cattle [40]. The donkey genome’s intergenic and intronic regions were predominantly composed of DMRs with a minor fraction associated with the 5′UTR and 3′UTR. A significant discovery was the identification of DMGs primarily in the YD-vs.-MD and YD-vs.-OD group comparisons, with a minimal number in the MD-vs.-OD comparison. Heatmap clustering analysis of gene functional regions highlighted variances in gene expression across different developmental stages, suggesting age-related changes in DNA methylation patterns within these regions that could influence gene expression. This hypothesis is supported by parallel findings in human studies [41]. This research posits that variations in gene expression within the upstream 2 Kb (up2k) and gene body regions may be attributed to decreased methylation levels, thereby enhancing the transcriptional activity of genes during the YD stage. Conversely, the downstream 2 Kb (down2k) region exhibited reduced gene expression at the YD stage, potentially due to increased methylation, which could impede gene expression. A comprehensive functional enrichment analysis was conducted on the DMGs across three comparative groups. Notably, in the YD-vs.-MD and YD-vs.-OD groups, an array of skin collagen-related pathways was identified within the KEGG pathway analysis, including ECM–receptor interaction, the relaxin signaling pathway, the MAPK signaling pathway, and the Hippo signaling pathway. However, such pathways were not discernible in the MD-vs.-OD group, likely due to the limited number of DMGs, precluding their detection in the functional enrichment analysis.

The study unveiled multiple genes integral to the deposition and degradation of collagen within the skin, encompassing *COL1A1*, *COL1A2*, *COL3A1*, *COL4A1*, *COL4A2*, *GLUL*, *SFRP2*, *FOSL1*, *SERPINE1*, *MMP1*, *MMP2*, *MMP9*, and *MMP13*. These genes were predominantly enriched in pathways such as ECM–receptor interaction, the relaxin signaling pathway, the MAPK signaling pathway, and the Hippo signaling pathway. The genes *COL1A1* and *COL1A2* are pivotal for synthesizing type I collagen, fundamental to the structural integrity of the body [42]. The *COL3A1* gene, responsible for type III collagen deposition, is vital for skin’s structure and function [43]. Type IV collagen, encoded by the *COL4A1* gene, plays a crucial role in wound healing and embryogenesis [44]. Overexpression of FOSL1 affects cell adhesion to fibronectin and collagen, impacting cell cycle progression [45]. *SERPINE1* contributes to fibrotic adhesions in injured flexor tendons by inhibiting MMP activity [46]. The matrix metalloproteinases (MMPs) family, including *MMP1*, *MMP2*, *MMP9*, and *MMP13*, degrades various extracellular matrix (ECM) protein substrates, including collagen [47]. Overexpression of *MMP1* leads to the degradation of ECM components, affecting the normal structure of collagen and elastic fibers, which may result in skin aging manifestations such as wrinkles [48].

This investigation revealed that the expression of collagen-related genes in donkeys is modulated by DNA methylation patterns. Specifically, the *COL1A1* gene exhibited the lowest average methylation level during the YD period, differing significantly from the MD and OD periods. The reduced methylation level of the *COL1A1* gene during the fetal period might be necessitated by the demand for heightened gene expression to support fetal growth and development, a phenomenon also observed in humans [37]. The methylation level of the *MMP9* gene remained consistent across the three periods, suggesting that methylation might not be the primary factor influencing *MMP9* gene expression in collagen deposition and degradation. Other epigenetic mechanisms, such as histone modifications and transcription factor regulation, may also play roles [49].

Although the pivotal role of *COL1A1* in collagen deposition is well documented, the specific regulatory mechanisms, particularly the role of promoter DNA methylation in varying developmental periods of donkey skin collagen deposition, remain elusive. Future research endeavors will aim to explore the functionality of potential genes in collagen deposition within donkey skin more comprehensively. Additionally, there is a need to elucidate the variations in DNA methylation of regulatory elements associated with crucial genes involved in collagen deposition and deposition, encompassing both in vivo and in vitro studies.

## 5. Conclusions

In conclusion, this research provides a detailed DNA methylation atlas for Dezhou donkey skin, the first of its kind. By integrating DNA methylation data with transcriptomic analyses, the study identified a number of key genes potentially involved in the regulation of skin collagen deposition. These include *COL1A1*, *COL1A2*, *COL3A1*, *COL4A1*, *COL4A2*, *GLUL*, *SFRP2*, *FOSL1*, *SERPINE1*, *MMP1*, *MMP2*, *MMP9*, and *MMP13*. The findings from this study are expected to significantly contribute to advancing our understanding of the epigenetic mechanisms governing collagen deposition in Dezhou donkey skin at the genomic level.

## Figures and Tables

**Table 1:** Primers of methylation trial.

Primer	Sequence (5′-3′)	Production
*COL1A1*-CpG1-F	TTTTATTAAGATGGTATAAAAGGGG	163 bp
*COL1A1*-CpG1-R	ATATCTAAACCCTAAACATATAAACTCTT	

**Table 2:** The summary of data generated by genome-wide bisulfite sequencing.

Sample	Raw Data (bp)	Clean Data	Clean Data (%)	Mapped Ratio (%)	Q20 (%)
YD-1	72,478,223,100	70,905,443,678	97.83%	90.49	96.9
YD-2	83,599,930,500	81,884,673,906	97.95%	89.94	96.49
YD-3	81,479,674,800	79,816,131,823	97.96%	90.41	96.87
MD-1	75,431,070,600	73,914,308,509	97.99%	90.29	96.7
MD-2	80,349,082,500	78,746,354,773	98.01%	90.22	96.64
MD-3	77,751,545,400	75,957,186,447	97.69%	90.12	96.25
OD-1	79,657,478,400	77,820,858,668	97.69%	89.57	95.91
OD-2	75,182,412,600	73,607,402,411	97.91%	89.69	96.07
OD-3	72,051,872,400	69,294,945,450	96.17%	89.24	96.26

## Data Availability

All the data are available in the manuscript, however, if any additional information is needed, it will be provided on demand.

## Supplementary Materials

The following supporting information can be downloaded at: https://www.mdpi.com/article/10.3390/ani14081222/s1, Figure S1: DNA methylation trends upstream and downstream of genome-specific elements in every sample; Figure S2: The frequency distribution histogram of the distance from DMR to CGI and the distance from DMR to TSS; Figure S3: The figure of differentially expressed genes (DEGs) overlapped with differentially methylated genes (DMGs) and the figure of integrated analysis of DNA methylation levels and gene expression levels; Table S1: Statistical table detailing the methylation levels of C bases across different gene regions.
