# Supplementary material for: Elucidating the Role of Transcriptomic Networks and DNA Methylation in Collagen Deposition of Dezhou Donkey Skin
Source: Animals (Basel). 2024 Apr 18;14(8):1222. doi: 10.3390/ani14081222 (PMC11047689; doi:10.3390/ani14081222)
Supplement: Supplementary file 1 [file animals-14-01222-s001.zip › Supplementary Figure.pdf]

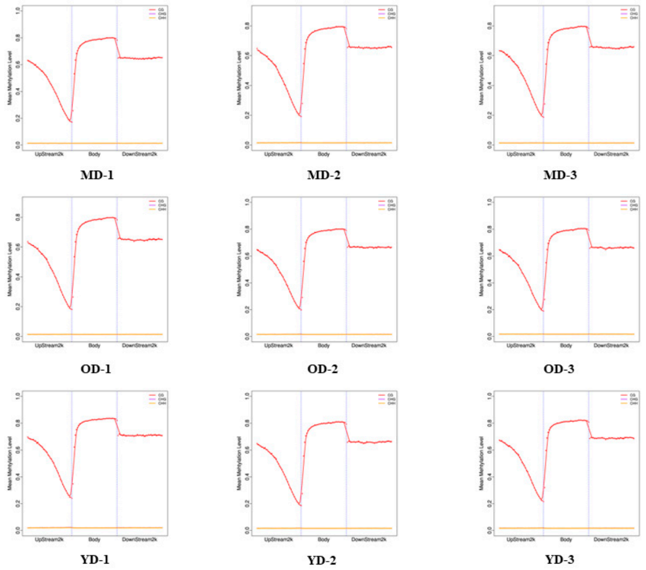

Supplementary Figure S1. DNA methylation trends upstream and downstream of genome-specific elements in every sample.

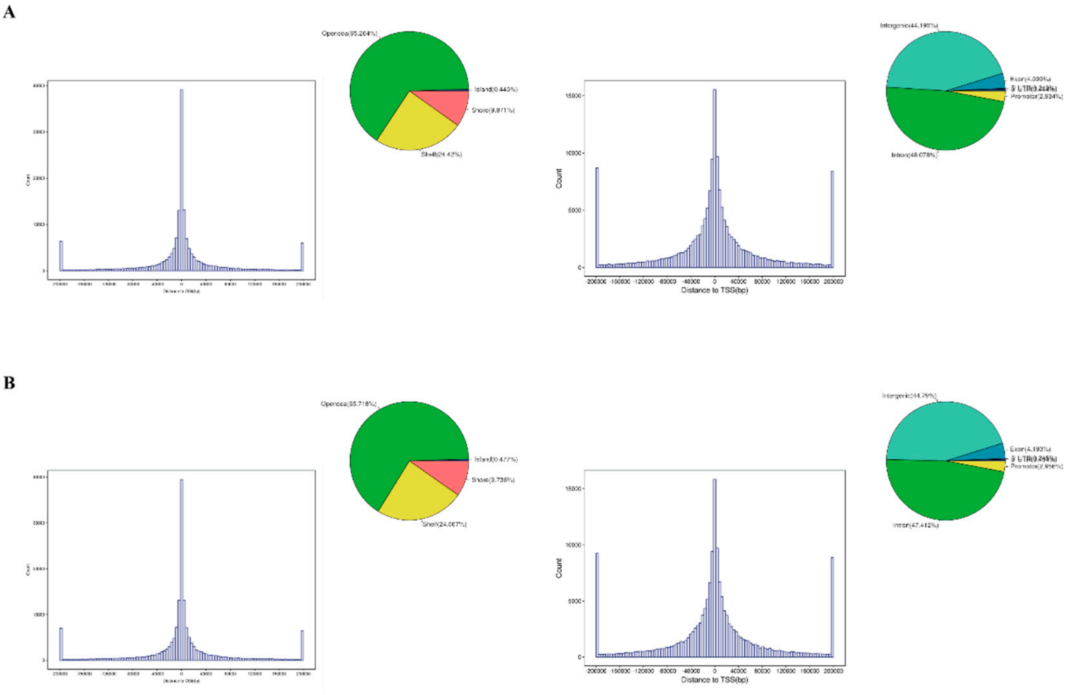

Supplementary Figure S2. The frequency distribution histogram of the distance from DMR to CGI and the distance from DMR to TSS. The DMR Annotation in CGI function elements (Island, Shore, Shelf and Opensea) and genome functional regions (5'UTR, 3'UTR, Exon, Intron, Promoter, Intergenic). (A) YD-vs-MD group. (B) YD-vs-OD group.

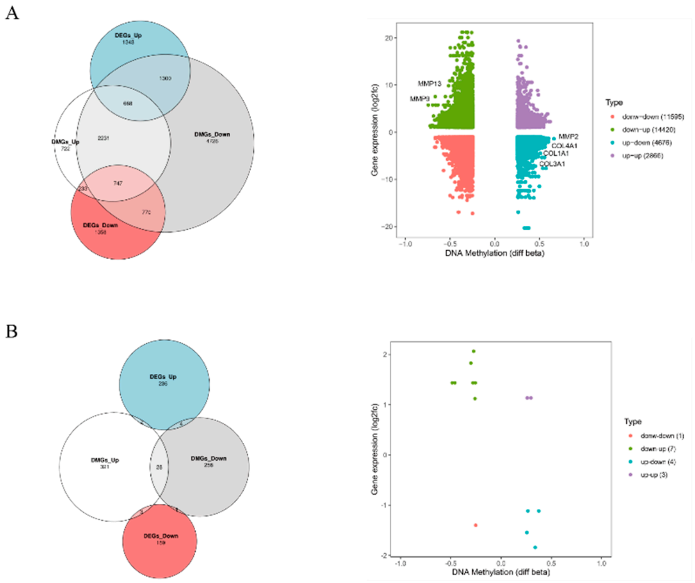

Supplementary Figure S3. The figure of differentially expressed genes (DEGs) overlapped with differentially methylated genes (DMGs) and the figure of integrated analysis of DNA methylation levels and gene expression levels. (A) YD-vs-MD group. (B) MD-vs-OD group.
